# The structure of human apolipoprotein C-1 in four different crystal forms

**DOI:** 10.1194/jlr.M089441

**Published:** 2018-12-17

**Authors:** Alexander McPherson, Steven B. Larson

**Affiliations:** Department of Molecular Biology and Biochemistry, University of California, Irvine, CA 92697-3900

**Keywords:** cholesterol trafficking, molecular imaging, X-ray crystallography, plasma lipid transfer proteins, atherosclerosis, cholesterol metabolism, HDL, LDL, lecithin cholesterol acyltransferase

## Abstract

Human apolipoprotein C1 (APOC1) is a 57 amino acid long polypeptide that, through its potent inhibition of cholesteryl ester transferase protein, helps regulate the transfer of lipids between lipid particles. We have now determined the structure of APOC1 in four crystal forms by X-ray diffraction. A molecule of APOC1 is a single, slightly bent, α-helix having 13–14 turns and a length of about 80 Å. APOC1 exists as a dimer, but the dimers are not the same in the four crystals. In two monoclinic crystals, two helices closely engage one another in an antiparallel fashion. The interactions between monomers are almost entirely hydrophobic with sparse electrostatic complements. In the third monoclinic crystal, the two monomers spread at one end of the dimer, like a scissor opening, and, by translation along the crystallographic *a* axis, form a continuous, contiguous sheet through the crystal. In the orthorhombic crystals, two molecules of APOC1 are related by a noncrystallographic 2-fold axis to create an arc of about 120 Å length. This symmetrical dimer utilizes interactions not present in dimers of the monoclinic crystals. Versatility of APOC1 monomer association shown by these crystals is suggestive of physiological function.

Apolipoprotein C-1 (APOC1) is a 57 amino acid polypeptide of Mr = 6.6 kDa (1) that is synthesized predominantly in the liver of mammals. Its gene product includes a 26 amino acid long signal peptide that is proteolytically removed in the rough endoplasmic reticulum upon secretion. In humans, the plasma concentration is about 6 mg/dl. This small protein is remarkable for being the most positively charged protein in the human body, with a lysine content of 17%. The polypeptide contains no histidine, tyrosine, or cysteine, nor is it attached covalently to any carbohydrate (1, 2). The protein has been the subject of numerous structural studies by NMR (3–5) and other biophysical techniques, but no crystal structure, to our knowledge, has been reported.

In human serum, APOC1 is responsible for the activation of esterified lecithin cholesterol. It plays an important role in the exchange of esterified cholesterol between lipoprotein particles and in the removal of cholesterol from tissues. The protein has a high affinity for lipid surfaces, particularly chylomicrons, and both HDL and VLDL particles. In the fasting state, APOC1 is mostly associated with HDL, but in the fed state, it redistributes to the surfaces of chylomicrons and VLDL particles. As a consequence, the presence of APOC1 on a lipoprotein particle may prolong its residence time in the circulation and subsequently facilitate its conversion to LDL (2, 6).

APOC1 had long been known to be a crucial player in the transfer of lipids between particles and the general clearance of lipids (2), but its mechanism and the manner by which it bound to lipoprotein particles was poorly understood (5). It was then discovered (7, 8) that APOC1 is a potent inhibitor of cholesteryl ester transferase protein (CETP), which is the crucial enzyme in the transfer of cholesterol between lipid surfaces (tissues) and lipid particles. It is this function, as an inhibitor of CETP, that likely explains its ubiquitous role in lipid metabolism. It is postulated that CETP inhibition by APOC1 may operate through the ability of APOC1 to modify the electrostatic surfaces of lipoprotein particles to which it binds (2, 9).

Because of its importance in lipid and cholesterol metabolism, APOC1 naturally plays an important role in medicine (10), particularly in atherosclerosis and other cardiovascular conditions (11), obesity (12), and diabetes (13). It would not be appropriate to review the field here, as an extensive literature on lipoproteins in medicine exists. It is, nonetheless, interesting to note that reports have appeared (14–16) showing that APOC1 is also associated with the hepatitis C virion. This suggests that its importance in medicine may be even more profound than previously understood.

About 25 years ago, we crystallized, for X-ray diffraction analysis, human APOC1 (17), using 2-methyl-2,4-pentanediol (MPD) as precipitant along with a nonionic detergent, octyl-β-d-1-thioglucopyranoside in the mother liquor. We ultimately grew crystals in four different crystallographic unit cells. Their properties are presented in **Table 1**. Two of the crystals, Monocln-1 and Monocln-2, are close variations of one another, the latter a less hydrated form of the former. The crystals, aside from these two, include another distinct monoclinic form, Monocln-3, and one orthorhombic form, designated Orthrhmb. All of the monoclinic crystals diffracted to at least 2.0 Å resolution, whereas the orthorhombic form diffracted to about 3 Å.

**TABLE 1.** Crystal and unit cell parameters

Crystal	Space Group	*a*, Å	*b*, Å	*c*, Å	β^o^	*V*_m_	Solvent, %	Resolution, Å	Mol/a.u.
Monocl-1	P2_1_	31.05	47.60	34.18	95.74	1.87	34	1.8	2
Monocl-2	P2_1_	27.88	46.45	34.09	94.12	1.64	25	2.0	2
Monocl-3	P2_1_	35.45	49.06	37.67	105.24	2.41	49	2.0	2
Orthrhmb	P2_1_2_1_2_1_	34.46	53.70	71.20	90.0	2.50	51	3.0	2

a.u., asymmetric unit.

In the years 1994–1995, at least 20 X-ray diffraction datasets were collected from native crystals and putative heavy-atom derivatives. Unfortunately, at that time, none of the heavy-atom derivative trials proved useful in phasing, which is not surprising because the protein contained no histidine, cysteine, or tyrosine. In addition, in spite of our best efforts, we were similarly unsuccessful in obtaining a structure solution using molecular replacement. Thus, the X-ray data were archived, and the project was essentially shelved.

Two years ago, we retrieved the X-ray data collected 25 years ago, which remain quite respectable even now (see **Table 2**), from our archives and resurrected the APOC1 analysis. We benefitted this time, however, by the past 25 years of development of powerful new approaches, computers, and software tools for crystallographic analysis, particularly in the areas of molecular replacement (18–21) and maximum-likelihood refinement (22, 23). This time our efforts were rewarded, and we successfully solved the structures of all four of the crystal forms of 25 years ago; we report them here.

**TABLE 2.** Data collection, processing, and scaling: Monocl-1, Monocl-2, Monocl-3, and Orthrhmb

Crystal	Monocl-1	Monocl-2	Monocl-3	Orthrhmb
X-ray source	Rigaku RU-200	Rigaku RU-200	Rigaku RU-200	Rigaku RU-200
Detector	SDMS	SDMS	SDMS	SDMS
Mosaicity	0.45°	0.48°	0.45°	0.68°
S.G. prob.	0.819	0.848	0.647	0.442
Resolution	35.0–1.80 Å	34.0–2.0 Å	30.0–2.0 Å	15.0–3.0 Å
Outer shell	1.95–1.80	2.10–2.00	2.05–2.0	3.18–3.0
No. of unique Fs	7,315 (613)	5,912 (406)	4,949 (206)	2,687 (460)
CC 1/2	0.993 (0.640)	0.996 (0.567)	0.989 (0.622)	0.997 (0.733)
*R*_merge_	0.094 (0.457)	0.144 (0.656)	0.126 (0.583)	0.169 (0.491)
*R*_meas_	0.114 (0.721)	0.152 (0.842)	0.146 (0.751)	0.180 (0.619)
*R*_pim_	0.055 (0.315)	0.079 (0.417)	0.070 (0.350)	0.080 (0.369)
Completeness	85.2 (.760)	95.4 (97.3)	76.0 (32.5)	91.8 (56.3)
Multiplicity	7.2 (4.5)	6.4 (3.7)	2.5 (1.5)	7.1 (1.7)
*I*/sigma	6.7 (3.2)	4.5 (1.4)	3.7 (1.2)	5.5 (1.4)
S.G. prob, space group probability.				

## EXPERIMENTAL

Details of the preparation and crystallization of the protein were presented in an earlier paper (17). APOC1 was crystallized by sitting-drop vapor diffusion (24) using Cryschem plates (Hampton Research, Aliso Viejo, CA). Reservoirs were 16–18% MPD with 0.10 M sodium acetate and 0.25% octyl-β-d-1-thioglucopyranoside. Crystallization droplets were composed of equal amounts of 8 mg/ml protein in 0.02 M NH_4_HCO stock solution and reservoir solution. Crystallization was at room temperature. All of the crystal forms reported here appeared from these same crystallization droplets, although every drop contained but one crystal form.

Crystals were mounted by conventional means in 0.7–0.8 mm quartz capillaries (24), and X-ray diffraction data were recorded at room temperature using a Rigaku RU-200 generator fitted with a Supper graphite crystal monochromator and operated at 40 kV and 30 mA with twin San Diego Multiwire Systems (SDMS) detectors at a crystal to detector distance of 420 mm. Images were processed with software provided by SDMS (25). Structure amplitudes were obtained by scaling and merging intensities from archived .ARC files using the program AIMLESS (26, 27) to yield comprehensive datasets (Table 1).

Molecular replacement searches were carried out using the program PHASER (18–20). Rebuilding and most graphics operations relied on the program COOT (28), as did quantitative comparisons of models. Refinement of the polypeptide models was accomplished using the program REFMAC (29) from the CCP4 program system (30) based on the maximum-likelihood approach (22, 23, 29).

## RESULTS

Shown in Table 1 are the cell parameters and solvent volumes of the four crystal forms with which we worked. The first two crystal forms in Table 1, Monocl-1 and Monocl-2, are very similar in cell dimensions and have the same space group, P2_1_, the latter crystal being a somewhat dehydrated form of the first, having about 13% less volume. Monocl-3 is also of space group P2_1_, but is otherwise unique, and it has significantly greater solvent volume, about 30% more. The last crystal form, Orthrhmb, is clearly different, having an even higher solvent content, about 32%, but it suffers from a limited resolution.

In our earlier efforts, we tried to determine a structure for any and all of these crystals, but concentrated primarily on Monocl-1 and Monocl-3. With no progress being made using heavy atoms, we worked to find solutions using molecular replacement. NMR studies of APOC1 indicated that the protein probably consisted of an amino-terminal α-helix and a carboxy-terminal α-helix that, through a hinge sequence near the center, allowed the two helices to form an intramolecular, antiparallel duplex (3, 4). All of our crystal forms contained two molecules of APOC1 as the asymmetric unit, suggesting further that APOC1 might exist as dimers. These considerations, along with some others, implied that the arrangements of antiparallel helices likely existed in solution, and in our crystals, as four-helix bundles, a common motif in proteins. The best available models for such an arrangement, using molecules of comparable size, were the ROP protein (31) and later the ROM protein (32). Neither, however, had functions similar to APOC1 or were otherwise related. As already noted, molecular-replacement attempts with these probes did not yield unique solutions where side-chain features of APOC1 could be recognized, nor could they be convincingly refined.

Without going into unnecessary detail, we recently resumed extensive molecular-replacement efforts using the program PHASER (18–20) from the CCP4 program package, again using four-helix bundle models and as many reasonable variations as we could conceive. All failed. Eventually we deduced, based mainly on intuition combined with observations on difference Fourier maps of our failures, that the APOC1 molecule was not composed of two antiparallel helical segments, but was a single, continuous helix along its entire length. With that realization, when polyalanine α-helices were properly placed, amino acid side chains appropriate to the sequence of APOC1 began to appear, and ultimately the entire APOC1 molecule emerged. Its correctness in Monocl-1 was confirmed not only by the appearance of the amino acid side chains, but by its refined agreement between calculated and observed structure amplitudes and by its use in solving, by molecular replacement, the other three crystal forms. The final refinement statistics for the structures of all crystals are presented in **Table 3**.

**TABLE 3.** Refinement and model

Crystal	Monocl-1	Monocl-2	Monocl-3	Orthrhmb
No. of Fs used	6,965	5,585	7,730	2,538
*R*_free_ test set	350 (4.82%)	404 (5.11%)	532 (5.12%)	130 (4.59%)
*R*_working_	0.2181	0.2333	0.2385	0.2025
*R*_free_	0.2770	0.2888	0.3096	0.2728
Mean B factor	19.0	22.9	25.2	62.5
RMS bond Δ	0.0103	0.0102	0.0091	0.0064
RMS angle Δ	1.22°	1.24°	1.30°	1.03°
RMS chrl. vol. Δ	0.065	0.070	0.074	0.058
Rama outliers	1: C terminus	0	0	0
Rotamer outliers	0	0	0	0
Twinning	No	No	Yes: 0.15%	No
TLS	No	No	No	No
Resolution, Å	1.75	2.0	2.0	3.0

chrl. vol., chiral volume; RMS, root mean square; TLS, translation, libration, screw.

### Crystal Monocl-1

**Fig. 1.**
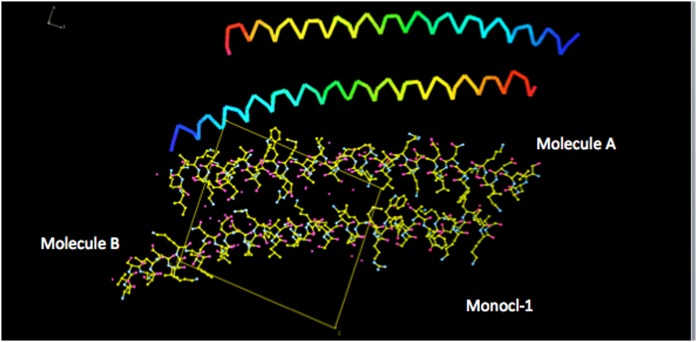
The structure of human APOC1 derived from the crystals designated as Monocl-1. At the top is a Jones’ rainbow representation of the courses of the polypeptide backbones of the two, virtually identical, monomers A and B making up the dimer of the crystallographic asymmetric unit. The amino termini are indicated by blue and the carboxy termini by red. The main chain of a monomer is a single, unbroken, but bent, α-helix of 13–14 turns. It has a length of 75–80 Å. At the bottom, in the same orientation, is an all-atom model of the dimer, with associated waters. The *a* × *c* plane of the Monocl-1 crystals is indicated.

**Fig. 2.**
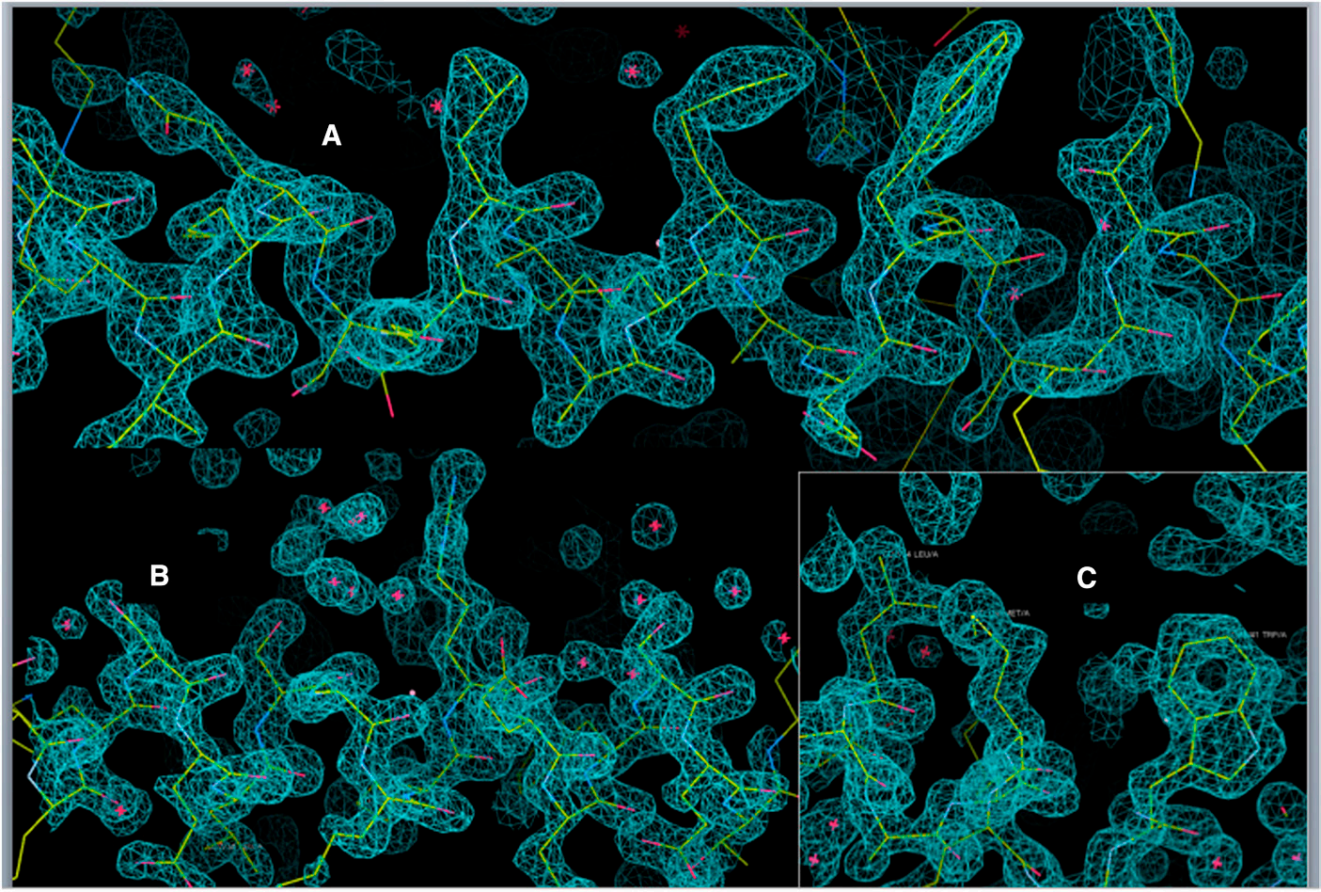
A: A central portion of one of the helices comprising an APOC1 monomer superimposed upon its electron density. The goodness of fit is evident and is typical of that for the entire structure. B: A similar illustration of electron density superimposed upon the model for the structure derived from crystals Monocl-3. C: A detail showing goodness of fit, featuring, at the right, the side chain of Trp41.

The structure of the asymmetric unit of the crystals designated Monocl-1 is shown in **Fig. 1**, and some details showing the quality of its electron-density maps are found in **Fig. 2**. The structure consists of two basically identical long α-helices, closely associated with one another and aligned in an antiparallel fashion. The α-helices are 52–55 amino acids long, with some residues at the termini being apparently disordered in some crystal forms and not visible. The helices are about 75 Å long and have 13–14 helical turns. The helices are not completely straight but are somewhat bent so as to better engage one another and enhance stereochemical complementarity of side chains. The structure is clearly a natural dimer; it is not a consequence of crystal packing. The entire dimer, given the overlap of the monomers, is about 95 Å long. The total buried surface area on the two monomers comprising the dimer is 2,595 Å^2^, or about 15% of the total surface area (18,355 Å^2^) of the dimer (program PISA; CCP4).

**Fig. 3.**
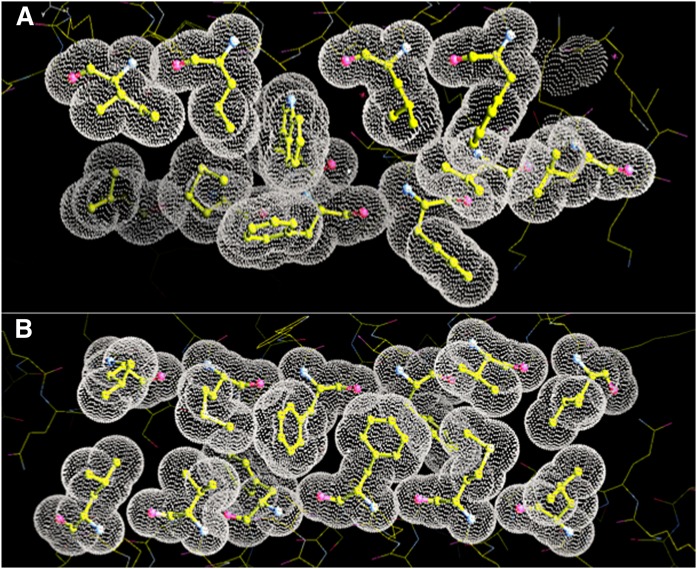
A: A van der Waals dot surface superimposed upon a stick-and-ball representation of the amino acid residues that comprise the hydrophobic interface between two monomers at one end of the APOC1 dimer in crystal Monocl-1. The top, horizontal stack of hydrophobic residues, from left to right, reads Ile29-Leu25-gap-Leu18-Phe14-Leu11; the register of the apposed bottom stack reads, from left to right, Leu34-Met38-Phe42-Trp41-Phe46-Val49-Leu53. B: The same rendering of the hydrophobic interface between the two monomers, at the dimer center, for the dimer in the orthorhombic crystal. The top horizontal stack of amino acid residues reads, from left to right, Leu34-Met38-Phe42-Phe46-Val49-Leu53. The bottom stack, symmetrically related by the NCS 2-fold axis, has the same sequence but read in the opposite direction. The very close packing of hydrophobic residues is evident at both interfaces.

On one monomer (A), on the amino-terminal half, there is a near-continuous stack of hydrophobic side chains. These are Leu11-Phe14-Leu18-**gap**-Leu25-Ileu29, with a gap between Leu18 and Leu25. On the other monomer, closely opposed to the stack on monomer A, is a different stack of hydrophobic side chains from the carboxy-terminal half of molecule B. This stack is Leu53-Val49-Phe46-**Trp41**-Phe42-Met38-Leu34. The two opposing hydrophobic stacks are not only in direct contact, but are further consolidated by the intercalation of part of the side chain of Trp41 into the gap of the opposite stack between Leu18 and Leu25. It is after Ile29 on monomer A and before Leu34 on monomer B that the two monomers begin to separate somewhat. This is illustrated in **Fig. 3A**.

In addition to the hydrophobic interface, there are also some possible salt bridges and hydrogen bonds between the two monomers, but these must be considered tentative, as they involve flexible side chains. They are Glu13A-Lys52B-Lys21A-Thr45B-Lys50A-Glu19B and Ser35A-Lys30B. A network of hydrogen bonds could exist involving Asp20B-Glu47A-Arg23B-Ser43A. Most of the possible hydrophilic interactions are at the opposite end of the dimer, where the two monomers begin to separate from where the hydrophobic stacks interact and hold the monomers closely together.

**Fig. 4.**
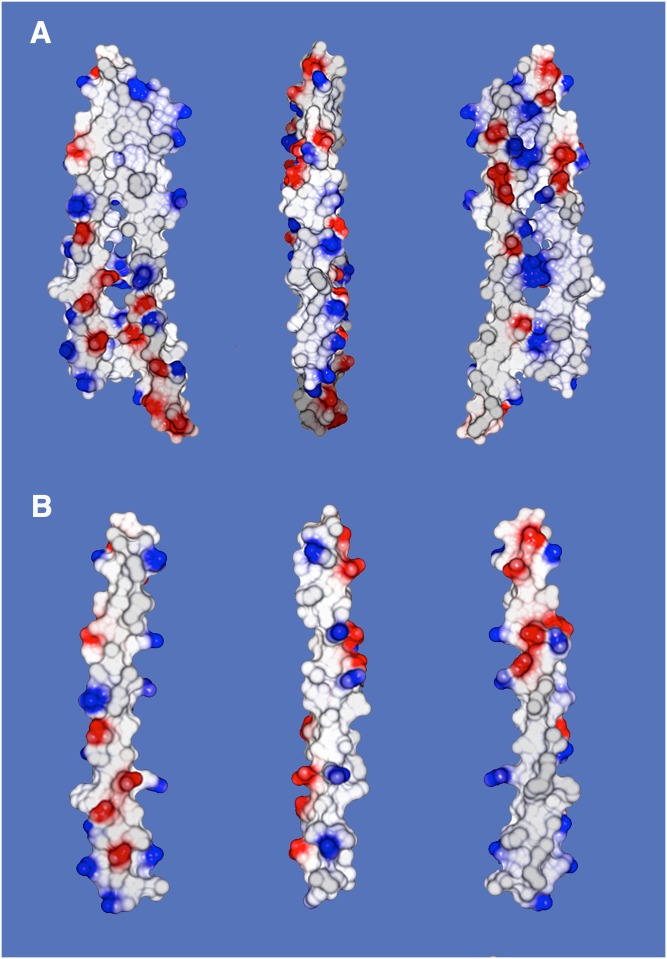
A: At left is an electrostatic surface representation of one face of the APOC1 dimer from Monocl-1. The top half of the dimer is characterized by a largely hydrophobic surface with only a few dispersed, mostly positively charged groups. The lower half, on the other hand, is richly populated with both positive and, even more, negatively charged groups. It is clearly hydrophilic. At the right in A, the dimer has been rotated a full 180°, and the characters of the top and lower halves are now reversed, with the top hydrophilic and lower hydrophobic. At center, the dimer is shown at midway, rotated by 90°. It simultaneously shows the unequal distribution of charges on the obverse surfaces. B: Similar to the dimer above, the electrostatic surface of an APOC1 monomer is shown in orientations related as 0°, 90°, and 180°, from left to right. In the isolated monomer, as was also true of the dimer, opposite faces exhibit distinctly hydrophobic and hydrophilic, charged halves. This is especially apparent in the monomer at 90° rotation.

Electrostatic surface renderings of the dimer, seen in **Fig. 4A**, reveal an interesting distribution of hydrophobic surface and charged groups. In the orientation at the left in Fig. 4A, it is evident that one-half of the molecule is largely hydrophobic, punctuated here and there by positively charged lysine residues, whereas the other half of the dimer exhibits a cluster of negative charges. Positive charges are, however, also present on that half as well. When the molecule is rotated 180° to show the opposite side, at the right in Fig. 4A, a similar surface is seen. Again, negative charge clustered within one half, the other half almost entirely hydrophobic, with positive charges scattered throughout. The areas of concentrated charge and hydrophobicity switch halves on the two sides of the dimer. This is further apparent in the center orientation in Fig. 4A, where the view is perpendicular.

### Crystal Monocl-2

The dimer from crystal Monocl-1, once known, was simply placed in the unit cell of Monocl-2 with the same coordinates; the two helices in the dimer were rigid body-refined and then subjected to restrained refinement (23, 33). The relevant refinement statistics are presented in Table 3. The individual monomers and the dimer appear virtually unchanged in the Monocl-2 crystals, but the two helices scissor slightly closer to one another by about 2 Å, the pivot being the hydrophobic cluster described above. The number of water molecules and their structure, as might have been expected from the volume change, are slightly different. Residues 5–8 are somewhat less ordered in Monocl-2 on one monomer, but on the other monomer, density is present for Val4, Asp3, and Pro2. These were not visible at all in the Monocl-1 crystal.

Although the movement of the two monomers within the dimer toward one another is slight, it significantly increases the interactions between them. The buried surface area is enlarged an additional 7% to 4,050 Å^2^ and becomes 22% of the entire surface area (19,021 Å^2^) of the entire dimer. Both the dimers of Monocl-1 and Monocl-2 are predicted by the program PISA (CCP4 System) to be stable dimers in solution.

Monocl-2 represents the dimer exhibiting the greatest degree of compression of the constituent monomer helices. As in Monocl-1, the hydrophobic stack interaction at one end of the dimer is fully maintained, but the opposite end of the dimer shows the two monomers pressed more closely together. This is consistent with the increase in buried surface area noted above. In Monocl-2, the hydrophilic interactions appear a bit more certain and involve Arg39A-Ser27B, Ser43A/Glu47A-Arg 23B, and Lys 50-Glu19B/Asn16B/Asp20B. There may again exist a network of hydrogen bonds. If so, this would involve Ser43A, Arg39A, Lys50A, Ser27B, Arg23B, Glu19B, and Asn16B.

### Crystal Monocl-3

Crystal Monocl-3 initially presented something of a challenge. It was indicated by the program POINTLESS (26, 27, 34) to have a twin fraction of about 0.15, according to the L test (35). When a molecular-replacement search (20) was carried out using the refined dimer from Monocl-1, no solution could be found. However, if a single monomer helix from Monocl-1 was used as a probe, a solution of two molecules of APOC1 within the asymmetric unit was quickly found, and it could be refined without difficulty. Although the individual monomers in Monocl-3 were essentially identical to those in Monocl-1, their arrangement in the crystal lattice, and the dimer they formed, was distinctly different.

**Fig. 5.**
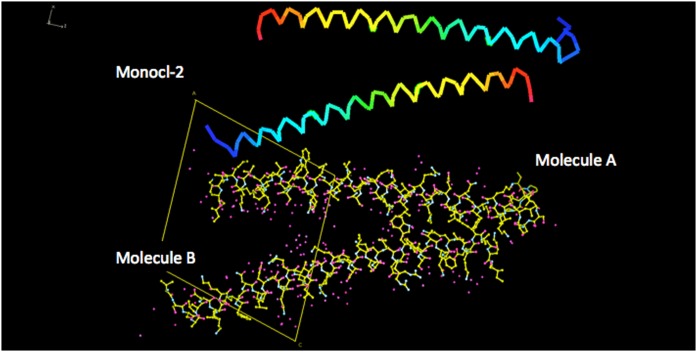
At the top is a Jones’ rainbow representation of the courses of the polypeptide backbones of the two, virtually identical, monomers of APOC1 comprising the asymmetric unit dimer of crystals Monocl-3. Blue indicates the amino termini and red the carboxy termini. At the bottom, in the same orientation, is an all-atom model of the dimer along with associated water molecules. The crystallographic *a* × *c* plane of Monocl-3 is indicated. It is important to note how the dimer seen in Fig. 1 (the dimer of Monocl-1) has spread apart at one end, while the opposite end provides a dynamic hinge.

The molecular-replacement solution for Monocl-3 appeared as two monomers lacking to some extent the clearly evident, intimate dimer relationship present in Monocl-1 and Monocl-2. The cluster of hydrophobic interactions at one end of the dimer of Monocl-1, although somewhat altered, appeared, however, to be largely preserved. The monomeric helices, however, do not exist in isolation, as seen in **Fig. 5**. Any one helix (A) is also closely associated with a second helix (B) related to the first by a translation along the crystallographic *a* axis. The occluded surface area and specific interactions between them are, in fact, quite extensive. Thus, two monomers once again form a dimer, but a dimer different, and less self-involved, than that of Monocl-1 and Monocl-2. There is, however, an interesting complexity. The two monomers A and B that make up a dimer contact not only one another, but, as noted above, form almost equally extensive interactions with the A and B molecules related by unit cell translations along the *a* axis. This means that the crystal is not made up of independent dimers, but that A/B dimers form continuous, and contiguous, bands or sheets of indeterminate length running through the Monocl-3 crystals

For the monomers of the dimer sharing the hydrophobic cluster, the buried surface area, as a consequence of total oligomer formation, on A is 1,229 Å^2^, and on B, it is 1,362 Å^2^, so that the total buried surface area within the dimer, 2,591 Å^2^, is about 13% of the total surface of 19,623 Å^2^. This is similar to the percentage of surface buried in the dimer of Monocl-1, but there is a crucial difference. In Monocl-3, the total buried surface area is shared between any one monomer A with B of the principal dimer (the dimer through which the most interactions, the hydrophobic interactions, take place between monomers) and with a second monomer (B) related to the first by a unit cell translation (35.45 Å) along the crystallographic axis *a*.

As expected from the volume increase, there is more solvent in the asymmetric unit of Monocl-3 than in Monocl-1 (49% vs. 34%); thus, the molecules are significantly more hydrated. This may, in fact, be the key to their rearrangement from the dimer of Monocl-1 to the more extended multimeric state that characterizes Monocl-3. Interpretable density is present beginning at Leu8 at the N terminus of one monomer, but there is disordered density that could accommodate the amino acid sequence all the way from Thr1. This portion of the polypeptide has been built, but with alternate chain conformations and must be considered tentative. On the other monomer, recognizable density begins at Val4.

In the dimer of Monocl-3, there are no longer any obvious hydrophilic interactions at one end of the dimer where the two monomers have sprung apart. At the other end, however, the two monomers still remain associated through the apposition of the hydrophobic stacks on the two monomers that were also present in Monocl-1 and Monocl-2. It does appear, however, that there is some perceptible rearrangement of the hydrophobic side chains when the dimer of Monocl-3 is compared with that of Monocl-1.

### Crystal Orthrhmb

The fourth crystal form, designated Orthrhmb, might expect less attention because its resolution limit is a relatively low 3.0 Å, but it is very interesting nonetheless for its uniqueness. The crystal is of space group P2_1_2_1_2_1_, has a unit cell volume that is greater than Monocl-3 (see Table 1), and clearly exhibits a different packing arrangement. Again, molecular-replacement searches using as a probe the refined dimer structure of APOC1 from Monocl-1 failed to provide any solution. As with Monocl-3, however, when the probe was a single helical monomer, a two-molecule asymmetric unit solution quickly appeared.

**Fig. 6.**
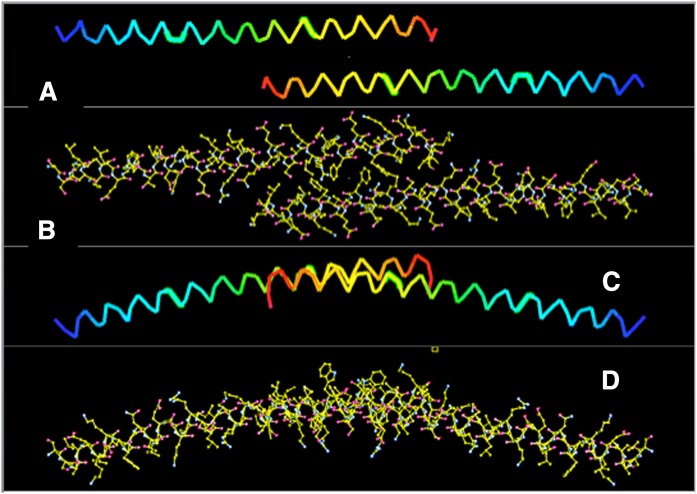
A, C: Jones’ rainbow representations of the polypeptide backbones of the two, virtually identical, monomers comprising the unique dimer that makes up the asymmetric unit of the orthorhombic crystals (Orthrhmb). Blue denotes the amino termini and red the carboxy termini. The views in A and C are related by a 90° rotation about the horizontal. B: An all-atom model of the dimer shown in the same orientation as its backbone in A. D: An all-atom model of the dimer in the same orientation as its backbone in C. Note particularly the different interface by which the monomers are associated in this dimer as compared with those in the monoclinic crystals. Note also the curved arc formed by the dimeric arrangement of the bent helices.

The structure of a unique dimer again emerged from the Orthrhmb crystals, but it was quite different than any of those seen in the monoclinic crystals. That dimer is shown in **Fig. 6**. Unlike the dimers in the monoclinic crystals, this dimer makes no substantive contact with others related by crystallographic symmetry or unit cell translations that would suggest higher oligomers. There is no apparent sheet formation as seen previously. A prominent feature of the dimer, evident in Fig. 6, is the presence of a noncrystallographic (NCS) 2-fold axis passing through the dimer between NCS equivalent Tyr41 side chains.

The two helices comprising the dimeric asymmetric unit of the orthorhombic crystals again share a large interface and exhibit many specific interactions. According to the program PISA (CCP4 System), the dimer clearly would exist as a stable unit in solution. The presence of the 2-fold axis is unique and is further supportive of its existence as a natural, stable dimer, even though it is the most hydrated form of APOC1 in any of the crystals. This dimer does not, as noted above, form any extended arrays. It stands alone as an independent dimer. Because the dimers in Orthrhmb crystals are unique and crystallographically independent of one another, all of the buried surface area belongs to the interface between the two monomers (A and B) within the dimer. The total buried surface area of the two monomers within the dimer is 2,601 Å^2^, or about 14% of their total surface area of 18,443 Å^2^. This is comparable to that for the dimers in the monoclinic crystals.

Although of lower resolution, the electron density was in general explicit, and amino acids could be readily recognized. At 3.0 Å resolution, placing water molecules is somewhat risky, but some were added where they appeared unambiguous. In this structure, density was clearly present at the N terminus on one monomer all the way from Pro2, but only from Ser5 on the other. In all of the structures, from all of the crystals, density at the carboxy terminus always ended exactly at Ile55. Amino acids Asp56 and Ser57 were never seen.

The interactions that maintain the dimer of Orthrhmb are obviously not the same as those that maintain the dimers in the monoclinic crystals, and these interactions are equally interesting. On one monomer (B), they utilize the same stack of hydrophobic side chains as was seen in the monoclinic crystals, Leu34-Met38-**Phe42**-**Phe46**-Val49-Leu53. On the other monomer (A), however, the stack utilizes some different side chains than in the monoclinic crystals. Because of symmetry, the stack must be the same, but running in the opposite direction. This stack is composed of Leu53A-Val49-**Phe46**-**Phe42**-Met38-Leu34. This contact area is shown in Fig. 3B. The interesting feature of the interface here is that the two stacks intercalate phenylalanine side chains into one another, so that each stack contains a core that reads **Phe46B**-**Phe42A**-**Phe42B**-**Phe46A**.

There does not appear to be any significant hydrophilic interactions that further contribute stability to this dimer. It depends almost entirely on interactions between the hydrophobic stacks. From the regularity of the spacings of the hydrophobic groups along the interface of this dimer, as well as the interfaces seen in the dimers of the monoclinic crystals, the amphipathic character of the helical monomers is pronounced.

## DISCUSSION

There are at this time three other apolipoprotein structures that have been determined by X-ray crystallography: they are apolipoproteins E (36), Protein Data Bank (PDB) ID 1LPE; and two structures of APOA-1, the major protein of HDL. The first of the APOA-1 structures is of a truncated protein lacking amino acids 1–43 (37), PDB ID 1AV1, and the second is of a mutant missing the segment consisting of amino acids 185–243 (38), PDB ID 3R2P. The crystallographic asymmetric unit in the first case was a tetramer of the protein and in the second a dimer. The difference in quaternary structure represented the greatest difference between the two, as the secondary and tertiary structures were essentially the same, a very long, “pseudocontinuous” amphipathic α-helix (5). The structures of APOA-1 were also analyzed by NMR (39), with little disagreement between the NMR and crystal structures and most differences attributable to the fact that the crystal structures were carried out in solution in the absence of lipids or detergents, whereas the NMR work was done in the presence of either sodium dodecyl sulfate (SDS) or dodecylphosphocholine (DPC). A comparison of the results of the two kinds of studies has been ably reviewed elsewhere (5).

The known crystal structures of all apolipoproteins, as well as all NMR analyses, show the apolipoproteins to be composed almost entirely of amphipathic α-helices, as in APOC1, sometimes long and continuous, almost always curved, sometimes segmented, and linked by hinges or short unstructured regions (5). The curvature of the helices tends to be an intrinsic property of long helices, and, in the case of amphipathic helices, produced by the relative solution environments on opposite sides of the helix.

It has been noted that there is considerable amino acid homology among the lipoproteins that suggests evolution from a common gene (40). Thus, it may be possible that APOC1 is simply a very truncated segment of an amphipathic α-helix present in another apolipoprotein, such as APOA1, and is useful in “filling in the gaps” left by the much larger APOA1 in HDL, for example. The structures of APOA1 have given rise to complex and intricate models for the development and ultimate form of HDL as reviewed by Gogonea (41). The inclusion of APOC1 may allow refinement of these models.

An important point, relevant to the investigation presented here is the medium in which the analysis was performed. Like the X-ray analyses of APOA1, this analysis also relied upon crystals grown from an aqueous solution, with no lipids present, but the mother liquor also contained 17% MPD as well as 0.25% octyl-β-d-1-thioglucopyranoside (17). The octyl-β-d-1-thioglucopyranoside is a nonionic detergent and may provide some of the character of a lipid environment, but certainly not that of the SDS or DPC used in the NMR studies. Thus, again, differences between the crystal structures of APOC1 and the NMR results may be attributable to the relative hydrophobic environments. On the other hand, it is pointed out that comparison of NMR and crystal structures of APO1 shows that in one region of 50 residues (50–101), the two analyses are in near agreement. This is in spite of differences in solution environment.

Specifically with regard to APOC1, the NMR structure of an N-terminal fragment of APOC1 was described as a single α-helix of about 38 residues (42), whereas determination by NMR of the intact protein indicated it to be two amphipathic helices linked by a short hinge region (4). In the NMR structure, the two helices folded upon one another, although as Cushley and Okon (5) point out, “the spatial orientation of the two helices with respect to one another is not well defined.” If the two helices are simply assumed to be collinear with the hinge region adopting an α-helical conformation (the amino acid sequence is compatible), then the NMR structure becomes virtually the same as the X-ray structure presented here. Indeed, both structures may exist, depending on the degree of hydrophobicity of the environment.

The opposing surfaces of an APOC1 monomer have a negatively charged half and a hydrophobic half, as seen in Fig. 4B. When the helix is rotated about its axis by 180°, a similar inverted distribution is seen. The half-hydrophobic and half-charged motif is preserved in the dimer, as was shown earlier by Fig. 4A. All surfaces are punctuated by a disperse array of positively charged lysine side chains, reflecting the pronounced positively charged nature of the polypeptide. We presume that the positive charges are present in order to interact through salt bridges with the negatively charged phosphate groups of phospholipids in lipoprotein particles.

**Fig. 7.**
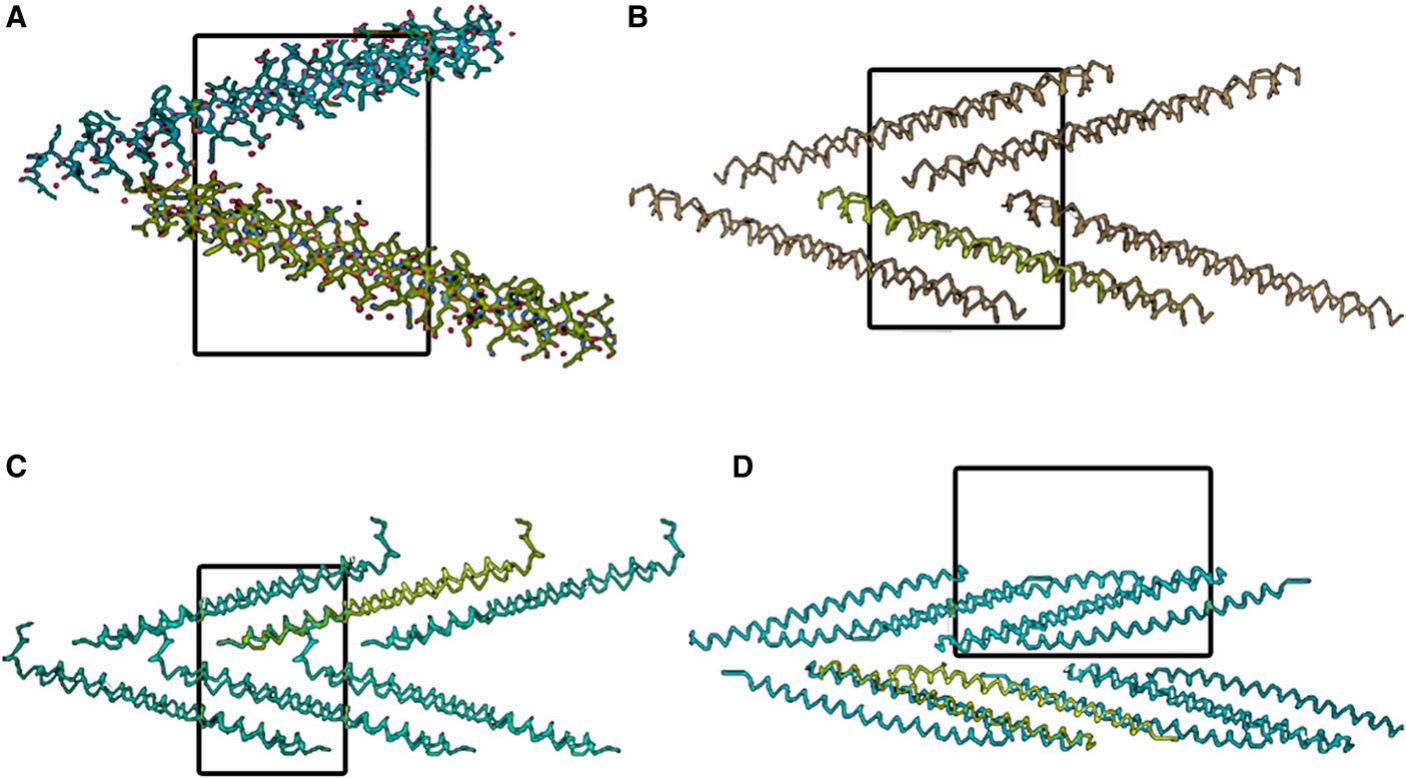
A: All-atom models of the dimers (four monomers) comprising the two asymmetric units, the entire contents, of a single crystallographic unit cell of the monoclinic crystals (space group P2_1_) Monocl-1. The view is exactly along the crystallographic *a* axis in A–D. B: A more extended packing diagram using polypeptide backbone representations only for Monocl-1. Corresponding packing diagrams are shown for Monocl-2 and Monocl-3 in C and D, respectively. As evident from these diagrams, APOC1 dimers pack along the crystallographic *a* direction to form infinite flat, zig-zag sheets throughout the crystals.

**Fig. 8.**
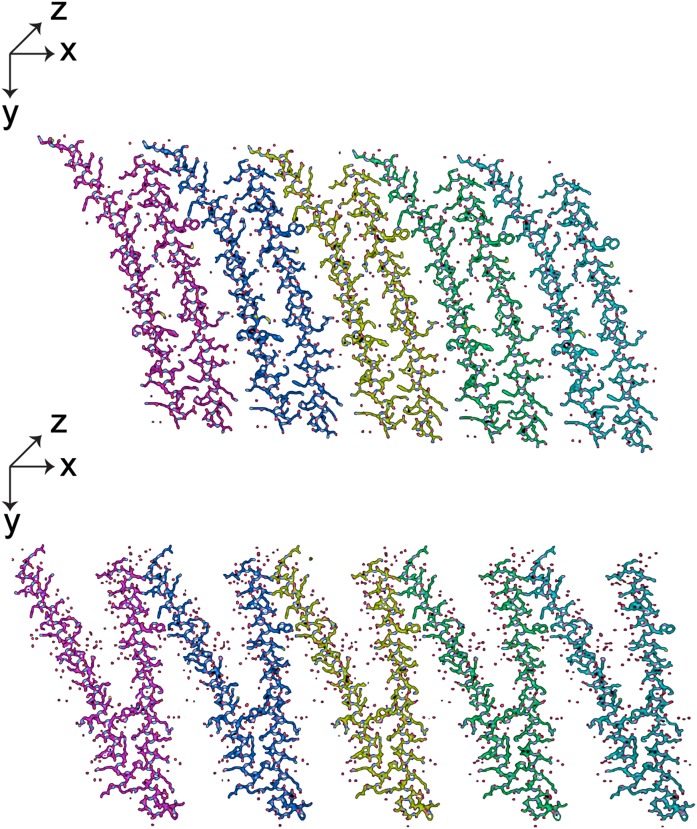
All-atom models of five consecutive APOC1 dimers, with Monocl-1 at the top and Monocl-3 at the bottom, form extended sheets along the *a* crystallographic direction, which is horizontal and in the plane of the figure. Note not only the intimate contacts between monomers at the lower end of each dimer, but also the substantive interactions at their opposite ends where dimers are related by unit cell translations.

**Figure 7** presents packing diagrams for Monocl-1, Monocl-2, and Monocl-3 using only backbone models. That for Monocl-2 is very similar to that for Monocl-1, with some slight compression of the two monomers together within the dimer, as described above. Evident from Fig. 7, when viewed along the crystallographic *a* axis of any monoclinic unit cell, asymmetric unit dimers form continuous, planar sheets. There is close association between dimers of one unit cell and those in the next unit cell related by translation along *a*, so that protein dimers contact one another in a contiguous manner along the sheets. This is true only along the *a*-axis direction and not for the *b*- or *c*-axis directions. If the linear arrays of dimers, or sheets, are viewed along the perpendicular, unique *b* axis, they have the appearance shown in **Fig. 8**.

**Fig. 9.**
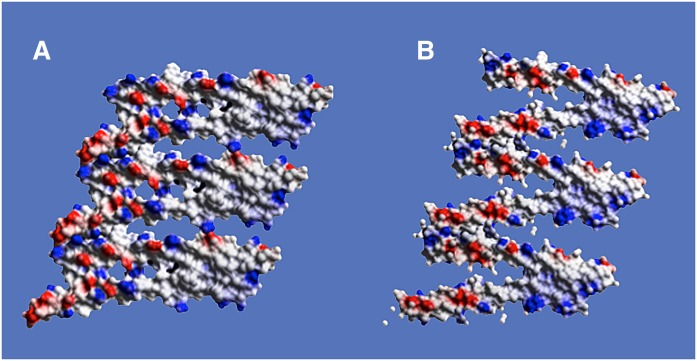
The sheets formed by simply translating an APOC1 dimer by **/a/** along the *a* crystallographic direction is shown as electrostatic surfaces. Only three dimers are included in these sheets, which are from Monocl-1 in A and Monocl-3 in B. The orientations are 90° to those in Fig. 8. Note that the hydrophobic/hydrophilic character of the two ends of the sheets, just as seen for a single dimer in Fig. 4A and an isolated monomer in Fig. 4B, is preserved and propagated.

In the less hydrated (34% solvent) Monocl-1 crystals, and the even less hydrated (25% solvent) Monocl-2 crystals, dimers are so closely packed, molecule A against B of translationally related dimers, that the sheet or band is extremely dense. In Monocl-3, however, the dimers of Monocl-1 and Monocl-2 have essentially “opened up,” by spreading or scissoring apart at one end, accordion or bellows style, while at the same time maintaining a firm association with dimers translationally related along *a*. Thus, the dense bands of dimers in Monocl-1 and Monocl-2 become the zig-zag, pleated bands of Monocl-3. An electrostatic surface representation of the extended sheets using three dimers from Monocl-1 and Monocl-3 is shown in **Fig. 9**.

The ability of APOC1 dimers to form compressed sheets (or bands) at low hydration and stretched, zig-zag bands at higher hydration suggests that the dimeric arrangements of two helical molecules are dynamic. Through an accordion-like mechanism, they can transform from a very dense band (as in Monocl-1 and -2) to a more open and extended band (as in Monocl-3). Although the band seen in Monocl-2 likely represents the compression limit (the monomers can get no closer to one another), it is not certain that the bands found in Monocl-3 are necessarily the limit of maximum expansion. The bands could possibly be stretched even further.

It is hypothesized that APOC1 alters the activity of CETP by binding to the surfaces of lipoprotein particles and altering either access to lipids by CETP or inhibiting the activity of CETP directly. The former explanation appears most in favor at this time. If that is true, then contiguous binding of APOC1 dimers to lipid-particle surfaces to produce patches or a continuous, shielding surface may be a mechanism. The elasticity of the bands would allow them to associate with particles of widely different dimensions and shapes, as is known to be the case. It would also allow the APOC1 dimer bands to remain associated with a particle as its diameter changed as a consequence of lipid addition or loss.

To pursue this idea a bit further, we observe that the extent of a sheet in the direction of propagation is about 25 Å/dimer for the compressed sheets of Monocl-2, or about 35 Å/dimer for the stretched sheets of Monocl-3. The width of the sheets would be fairly constant at about 100 Å. If one were to assume an HDL particle diameter of roughly 100 Å, consistent with current estimates (6), then it would require only about nine dimers arrayed as in Monocl-3 to completely surround the lipid particle.

**Fig. 10.**
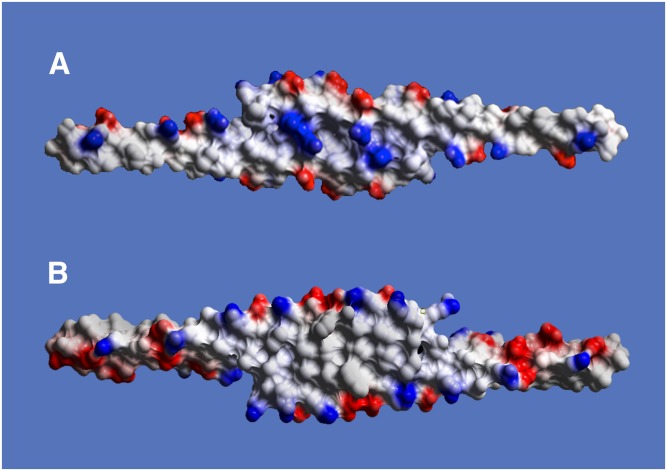
Viewed along the NCS 2-fold axis of the APOC1 dimer from the orthorhombic crystals in A, the concave side of the curved surface is hydrophilic and displays an array of positive charges that exhibit a rough spatial periodicity. B: The dimer has been rotated 180° to show the opposite convex side. This surface is hydrophilic at its ends, but exposes a large hydrophobic patch at the center of the dimer composed principally of the residues making up the hydrophobic interface shown in Fig. 4B.

Some interesting features of the orthorhombic crystal dimer, but whose significance we cannot judge at this time, are shown in **Fig. 10**. A noteworthy feature of the 2-fold symmetric dimer is the distinctive curvature (perpendicular to the NCS axis), evident in Fig. 6C, D. It is tempting to suggest that the concave surface might “fit” or “complement” the curved surface of a lipoprotein particle such as HDL. The distribution of charges on the concave and opposite convex surfaces, however, do not appear compatible with that idea. The concave surface seen in Fig. 10A exposes an array of positively charged groups exhibiting a regularity of spacing in traversing from one end of the dimer to the other. The positive charges crossing the surface are supplied by the symmetrical sequence of amino acids Lys10-Lys21-Arg28-(Lys50/Arg39)-(Arg39/Lys50)-Arg28-Lys21-Lys10. The spacings between them, beginning with Lys10-Lys21, are roughly 15 Å-10 Å-15 Å-10 Å-15 Å-10 Å-15 Å. The approximate periodicity is a consequence of the 2-fold axis perpendicular to the array and the distance between turns of the amphipathic helices bearing the charged side groups.

If the dimer is rotated by 180° to show the opposite, convex face, in Fig. 10B, then a completely different surface appears. There are concentrated charged groups at the extreme ends of the dimer, lines of periodically disposed positively charged groups that outline the top and bottom, but most striking is the large, completely hydrophobic surface that occupies the center. A rough estimate of its area is about 850 Å^2^. Were this area apposed to a similar hydrophobic area on another molecule or particle, it would imply a buried surface area of about 1,600 Å^2^. In protein terms, such an interface would be sufficient to imply the likelihood of a stable oligomer.

It may be relevant that in the structure of APOC2 deduced from NMR constraints, the amino-terminal helix (amino acids 16–36) also contains a pronounced bend. The unusual feature is that, like the APOC1 dimer from the orthorhombic crystals, the hydrophobic face of the amphipathic helix, which would be in contact with a lipid surface, is the convex face of the helix, not the concave face as might have been anticipated (43).

APOC1, the smallest member of the apolipoprotein family, is not significantly present in LDL, but is found in largest amounts in HDL (though APOA1 predominates) and is present in VLDL and chylomicrons as well. Thus, it is associated with particles that range in size from 4–10, 30–80, and 75–120 μm, respectively (6). Because the protein is embedded, or otherwise associated with particle surfaces, it likely assumes a variety of forms as the radius of curvature changes. That APOC1 dimers aggregate to form sheets that can expand or contract is what actually happens when APOC1 associates with lipid surfaces is, of course, speculative, but it is an attractive idea. It might even be possible to extend such assemblies in the direction of the helix axes to generate a much larger 2D sheet by joining the leading and trailing edges of the bands. This would require caution, however. In electron-density maps, amino acids Asp56 and Ser57 are never seen and therefore are likely disordered. Similarly, in several of the crystal forms, amino acids 1–4 are somewhat or totally disordered on some monomers. Therefore, extension of the bands into broad sheets by translation along the helix directions would require ordering and interaction of amino acids 1–4 of monomers with amino acids 56 and 57 of others. Such interactions would be difficult to predict.
